# Using Machine Learning to Predict Response to Inpatient Rehabilitation for FND Patients

**DOI:** 10.1192/bjo.2025.10449

**Published:** 2025-06-20

**Authors:** Amina Farah, Ayan Farah, Sheharyar Hassan Sheikh, Christopher Symeon

**Affiliations:** 1 St George’s University Hospitals NHS Foundation Trust, London, United Kingdom; 2 Cygnet Health Care, London, United Kingdom

## Abstract

**Aims:** Technology has been rapidly expanding in the medical field, of late, AI has been adopted cautiously and is slowly being integrated to practice. Functional Neurological Disorder (FND) patients have a variety of different presentations and premorbid conditions that greatly affect their response to rehabilitation. Currently, there is no admission formula or criteria available that can assist the assessing clinician on suitability for inpatient rehabilitation regarding rehabilitation prognosis.

The aim of this study is to design an admission formula using machine learning to predict rehabilitation prognosis; whether individuals with FND would benefit from inpatient rehabilitation by generating prognostic factors based off data collected from other FND patients who have received inpatient rehabilitation.

**Methods:** Retrospective review of FND patients admitted for inpatient rehabilitation. Over a 4-year period (2021–2024), 55 patients were admitted for FND neurorehabilitation, of which, 48 patients were used in the dataset due to lack of necessary data. Data was extracted from medical records and department databases to create a comprehensive dataset. The model was trained and tested by logistic regression, with a data set that was split into 70% training and 30% testing.

**Results:** The UK Functional Assessment Measure (UKFIM+FAM) was used to measure outcomes and patients were divided into two categories: improvement in FIM+FAM outcome above 25% from baseline or below. We discovered the model was 86% accurate in predicting the FIM+FAM outcome.

**Conclusion:** Machine learning may act as a tool that clinicians can use when assessing suitability for inpatient rehabilitation. Although there are limitations, namely, appropriate assessment scales and data-set size, the model is able to predict rehabilitation outcomes with 86% accuracy. Since this is supervised-learning, we expect with time and a larger data set, there will be improvement in accuracy.

